# An advanced fast method for the evaluation of multiple immunolabelling using gold nanoparticles based on low-energy STEM

**DOI:** 10.1038/s41598-024-60314-0

**Published:** 2024-05-02

**Authors:** František Kitzberger, Shun-Min Yang, Jiří Týč, Tomáš Bílý, Jana Nebesářová

**Affiliations:** 1 Laboratory of Electron Microscopy, Institute of Parasitology, Biology Centre CAS, 370 05 České Budějovice, Czech Republic; 2 Laboratory of Evolutionary Protistology, Institute of Parasitology, Biology Centre CAS, 370 05 České Budějovice, Czech Republic; 3 Faculty of Science, University of South Bohemia, 370 05 České Budějovice, Czech Republic; 4 Faculty of Science, Charles University, 128 00 Prague 2, Czech Republic

**Keywords:** Scanning transmission electron microscopy, High resolution scanning electron microscopy (HRSEM), Backscatter electron imaging, Simultaneous detection of multiple immunogold markers, Immunolabelling, Monte Carlo simulations, Electron microscopy, Structural biology, Data acquisition

## Abstract

We present a powerful method for the simultaneous detection of Au nanoparticles located on both sides of ultrathin sections. The method employs a high-resolution scanning electron microscope (HRSEM) operating in scanning transmission electron microscopy (STEM) mode in combination with the detection of backscattered electrons (BSE). The images are recorded simultaneously during STEM and BSE imaging at the precisely selected accelerating voltage. Under proper imaging conditions, the positions of Au nanoparticles on the top or bottom sides can be clearly differentiated, hence showing this method to be suitable for multiple immunolabelling using Au nanoparticles (NPs) as markers. The difference between the upper and lower Au NPs is so large that it is possible to apply common software tools (such as ImageJ) to enable their automatic differentiation. The effects of the section thickness, detector settings and accelerating voltage on the resulting image are shown. Our experimental results correspond to the results modelled in silico by Monte Carlo (MC) simulations.

## Introduction

The immune localization of biomolecules is based on the highly specific interaction between the antigen of the biomolecule and the antibody conjugated to the marker. This is the basis of a technique that has become integral to electron microscopy (EM) using EM visible markers conjugated to antibodies. The most frequently used markers are spherical gold nanoparticles (AuNPs)^1^. The AuNP tag possesses a high density and therefore can be visualized by electron microscopy (both transmission electron microscopy (TEM) and scanning electron microscopy (SEM)). Majority of the immune labelling studies require higher number of the biomolecules to be localized simultaneously in the same part of the specimen at the same time. For detecting two or more biomolecules simultaneously, two or more different markers must be used. This practically means using AuNPs with sufficiently different diameters. However, as antibodies with larger NPs have lower labelling efficiencies^2^, there is only a narrow range of NP sizes that can be used (ca 1–15 nm). Due to this, the labelling of more than two biomolecules is rather difficult. In the last 15 years, some solutions to this problem have been proposed. For TEM, AuNPs with various shapes, such as cubes, rods and tetrahedrons, were used. By combining different sizes and shapes of the marker, up to 5 epitopes could be detected simultaneously^3,4^. Another possibility is to use TEM equipped with an energy dispersive X-ray detector^5^ or energy-filtered TEM that should be able to detect NPs 3 nm in size^7^. However, a TEM microscope operating in STEM mode is able to distinguish NPs as small as 1 nm. When detecting signal from high-angle annular dark-field scanning transmission electron microscopy (HAADF-STEM), one can also detect and distinguish nanoparticles of different elemental compositions (Pt, Pd, Ag)^3,5,6^. This is also possible while using the detection of backscattered electrons (BSE) in high-resolution scanning electron microscopy (HRSEM), it is possible to distinguish AuNPs with different diameters and also NPs made from different materials due to the different greyscale levels/intensities of their imaging^8,9^. The biggest issue with the usage of the different shape/elemental composition of the markers is their availability. They have to be ordered unconjugated and their conjugation to given antibody is not straightforward.

The most recent technique for multiple immunogold labelling was suggested by Nebesarova et al.^10^ and uses SEM operating in STEM mode. This method is based on distinguishing Au nanoparticles located on the top and bottom of an ultrathin section labelled on both sides. Two images are acquired consecutively from the same area: the first image in STEM mode acquired with a high-energy beam (> 15 kV) shows Au markers at both sides, and the second image in BSE mode with a low-energy beam (< 1.5 kV) only at the top side.

Our study has improved this method so that only one accelerating voltage is used for the simultaneous imaging of AuNPs on both sides of an ultrathin section and the structure of the cell specimen. We show that this approach can be used for the localization of two biomolecules, gold immunolabelled by particles with the same diameter, thereby effectively doubling the number of targets that can be visualized.

## Results

**Figure 1:**
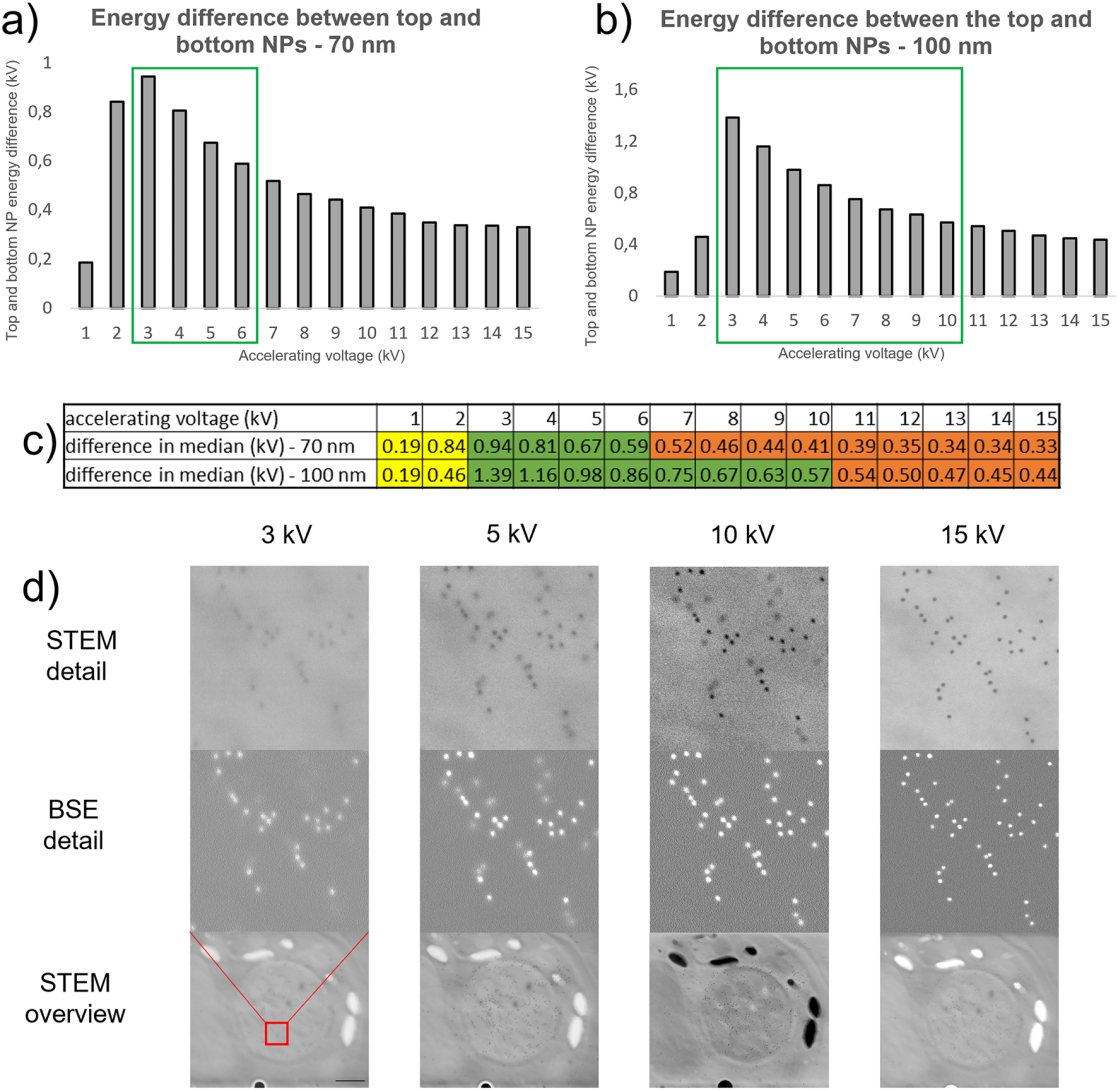
Selection of the optimal accelerating voltage for BSE and STEM imaging at one energy. (**a**, **b**) MC simulations of the difference between the BSE signals of the top and bottom AuNPs. The optimal range of accelerating voltages, according to the energy difference in the median, is highlighted in green. (**c**) Exact values of the differences in the median energies. The values in yellow belong to the accelerating voltages that do not penetrate the section, and the values in green represent the hypothetical optimal range for single-energy BSE imaging. The values in orange exhibit insignificant energy differences and therefore are not usable. (**d**) Rough visualization of NP (10 nm) visibility and distinguishability at different accelerating voltages on 100 nm sections with different settings of the STEM detector. Common parameters: resolution: 2560 × 1920, dwell time: 15.625 µs, scalebar at the overview image: 500 nm. STEM settings: 3 kV–B F + HAADF, 5 kV–BF + DF, 10 kV–DF, 15 kV–BF + DF.

The Monte Carlo (MC) simulation experiments in 2D were designed to confirm in silico the hypothesis that there is an energy difference of the BSE signal from the AuNPs on the bottom side of either the 70 or 100 nm ultrathin section and the ones on the upper side. The simulation set-up consisted of 10 nm layer of gold located above or below the 70/100 nm layer of acrylate. The simulation was run with 10,000,000 electrons focused to 2 nm spot. From the simulation results, we took the normalized distributions of the BSE energies from the Au layer, representing the AuNPs on the top or bottom, against the relative number of hits. The energy difference between the BSE signals of the top and bottom AuNPs was clearly visible from the peak representing the energy with the highest hit count (the most likely number of interactions of electrons with the resin); however, this accounted for less than 1% of the “detected energies”. Therefore, for statistical relevance, we decided to show the differences between the ranges of the highest hit count instead. For each accelerating voltage, we took the top 60% of signals with the highest hit counts, calculated their median, and used that to visualize the difference between the BSE signals of the top and bottom NPs. The full range of the distribution could not be used, as the signals were spread too much. The results of the simulations showed clear differences between the BSE energies of the signals of the top and bottom AuNPs and how they are related to the accelerating voltage used. The energy difference between the signals of the top and bottom AuNPs increased with decreasing accelerating voltage. The low difference at 1 and 2 kV was due to the beam not penetrating the section at all, and the BSE signal originated in the resin only and not in the gold below it. The hypothesis inferred from the results was that the optimal accelerating voltage lies between 3 and 6 kV for 70 nm sections or 3 and 10 kV for 100 nm sections (Fig. 1 a,b). At accelerating voltages larger than 6 kV or 10 kV respectively, the energy difference between the signals of the top and bottom nanoparticles is too low to provide a sufficient visual difference (Fig. 1 c).

This hypothesis was confirmed experimentally. The single accelerating voltage was sufficient to simultaneously visualize and distinguish the gold markers placed on both sides of the sections together with the cell structure. First, we used 100 nm sections with 10 nm AuNPs on both sides. At one selected area, multiple images at different accelerating voltages in the range of 3–15 kV (3, 5, 10, and 15 kV) were recorded and compared both with STEM and BSE imaging (Fig. 1d).

The most promising accelerating voltages—yielding sufficient observable differences between the signals of the top and bottom nanoparticles—were found to lie between 5 and 10 kV. The visual difference in the BSE images was visible at 5 kV; however, we also observed differences in the STEM images. The largest visual difference in the STEM images was at 10 kV, with the detector set to DF mode. In the BSE images, the top AuNPs are brighter and exhibit a sharp edge, while the bottom AuNPs have less contrast and have a blurry edge. In the STEM images, the top NPs have a darker shade of grey than the bottom ones. These visual differences are sufficient for the recognition of AuNPs lying on the top and bottom together with the specimen ultrastructure, which is visible in the STEM images (Fig. 1d).

**Figure 2:**
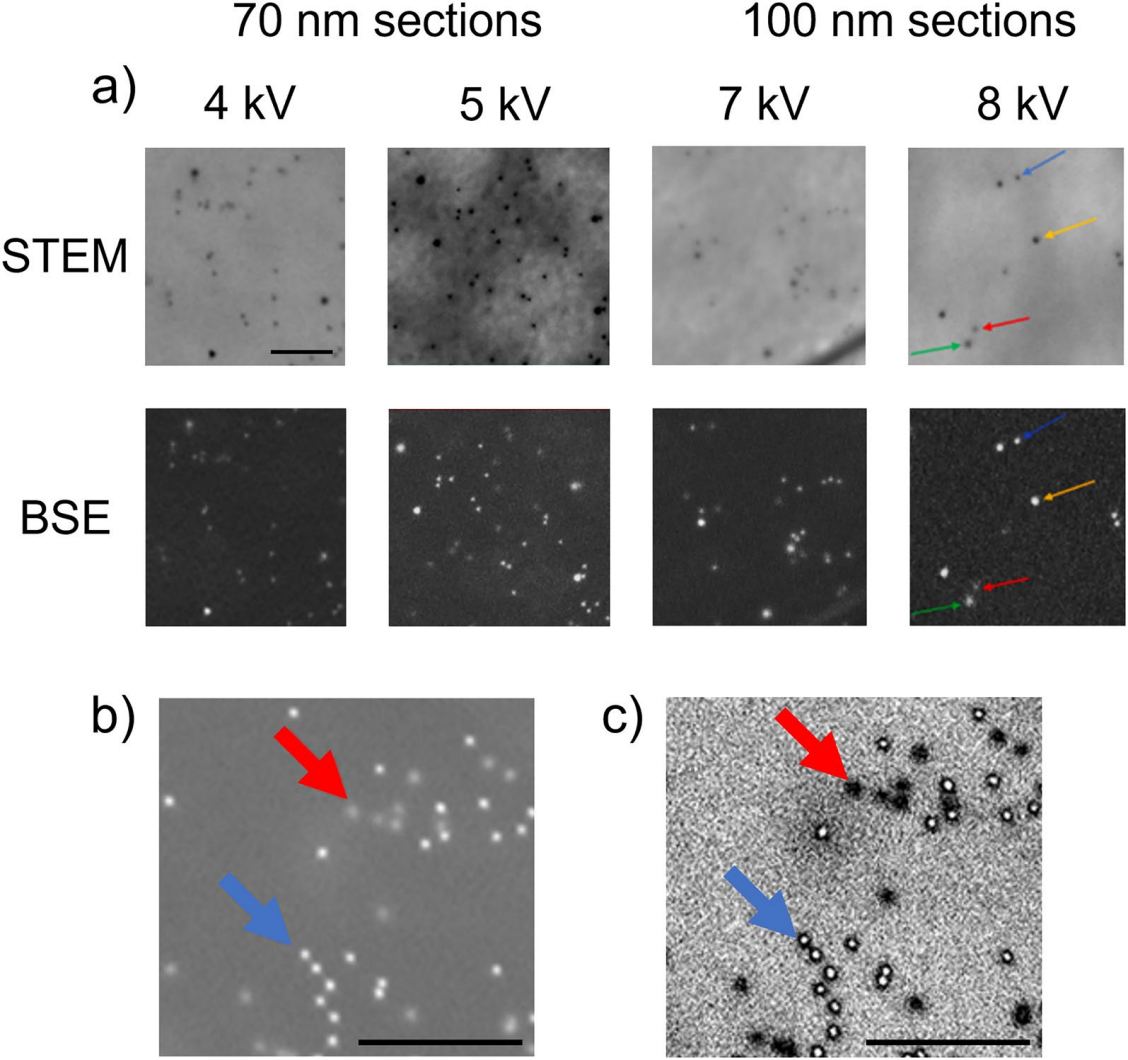
(**a**) Optimal parameters for the simultaneous imaging of the top and bottom markers using 10 and 15 nm AuNPs on both sides. Resolution: 6144 × 4096 px, dwell time: 10 µs, scale bar: 100 nm. Arrow colour coding: blue—10 nm NP on top, yellow—15 nm NP on the top, red—10 nm NP on the bottom, green—15 nm NP on the bottom; (**b**, **c**) software increased difference between the top and bottom 10 nm AuNPs in ImageJ. Colour coding: red arrow—bottom NP, blue arrow—top NP; (**b**) original BSE image. Scalebar: 250 nm resolution: 2560 × 1920, dwell time: 15.625 µs, working distance: 4.8 mm, 100 nm section, accelerating voltage: 5 kV. (**c**) ImageJ processed image—bottom NP—sphere shape, top NP—donut shape;

More accurate measurements (1 kV steps) determined that the most suitable accelerating voltage for the 100 nm sections is 7–8 kV. At this energy, the nanoparticles were visually well distinguishable in both BSE and STEM images, and the cellular structure was visible as well (Fig. 2). The STEM detector was operated with a combination of BF and DF.

When the recorded data were combined with those from the MC simulation experiments, the most suitable accelerating voltages produced an energy difference larger than 0.67 (Fig. 1c) and were powerful enough to penetrate the section. Therefore, the accelerating voltages selected for the 70 nm sections were those that fulfilled the same conditions: 5 kV and less.

The most suitable accelerating voltage for the 70 nm sections is 4–5 kV (Fig. 2.). The STEM detector operated with the BF + DF combination at 5 kV; however, at 4 kV, the STEM operated with the BF + HAADF combination, as it provided much better contrast and signal.

**Figure 3:**
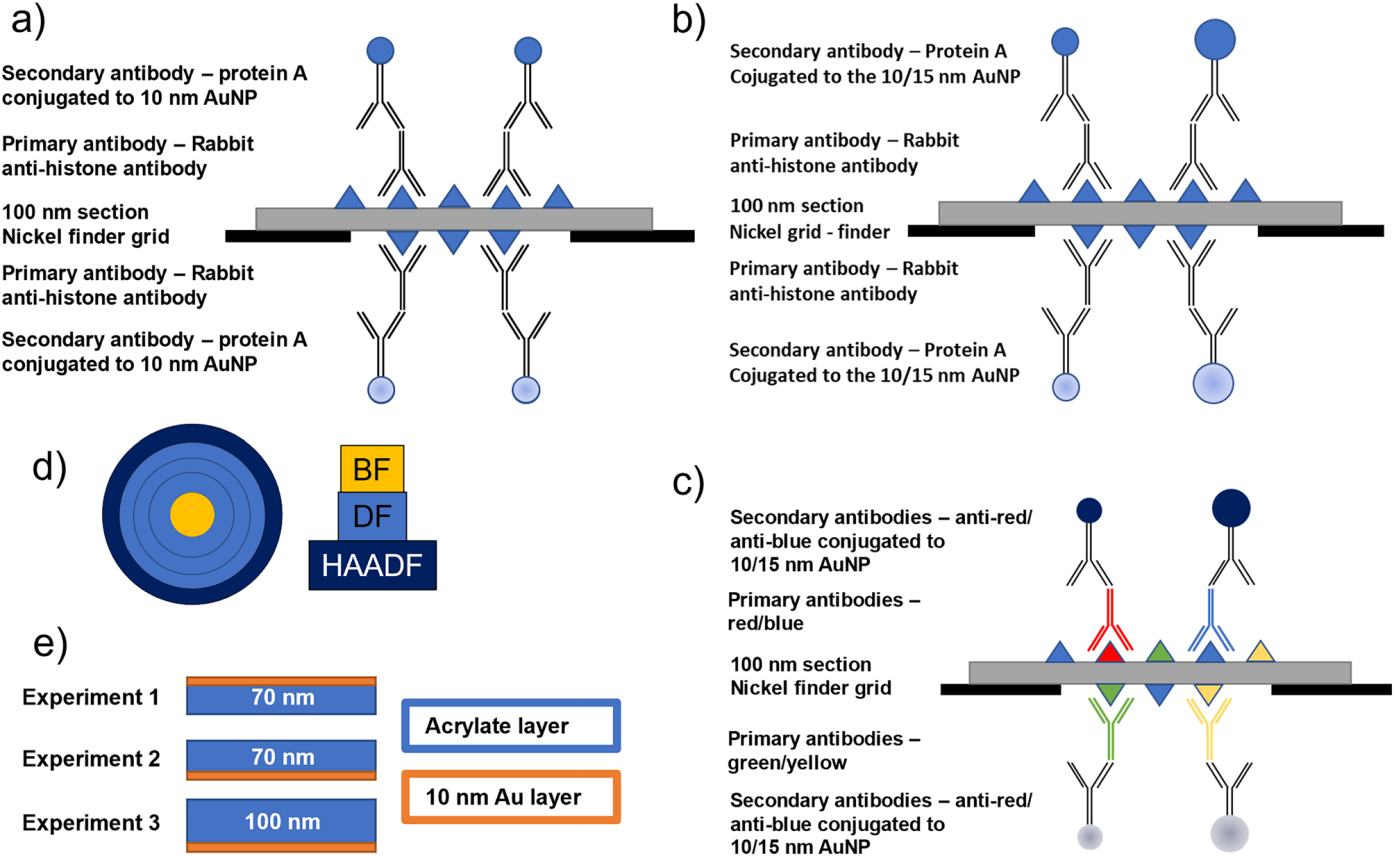
Illustration of the sample preparation and methods. (**a**–**c**) “immunolabelling” approach: (**a**) 10 nm AuNPs from both sides, (**b**) combination of 10 and 15 nm AuNPs from both sides, (**c**) possible approach for quadruple immunolabelling using 10 and 15 nm AuNPs; (**d**) scheme of annular STEM detector; (**e**) illustration of the material setup of Monte Carlo simulation experiments in Casino v2.51.

The detection and differentiation of four markers using just 2 AuNP sizes with the same imaging parameters (accelerating voltage corresponding to the section thickness) were also demonstrated. When 10 and 15 nm AuNPs were used as markers from both sides, we could differentiate 4 different markers, e.g., small and bright with a sharp edge (top 10 nm), small, less bright and blurry (bottom 10 nm), big and bright with a sharp edge (top 15 nm), big, less bright and blurry (bottom 15 nm) (Fig. 3). As mentioned above, the annular STEM detector was used to detect either the combination of BF (bright field) + DF (dark field) signals at higher voltages or BF + HAADF (high angle annular dark field) signals at lower voltages (Table 1).

**Table 1:** Optimal combinations/configurations of the STEM detector for a given section thickness and accelerating voltage based on visual observation.

Section thickness (nm)	Accelerating voltage (kV)	STEM detector settings
70	4	BF + HAADF
70	5	BF + DF
100	7	BF + DF
100	8	BF + DF

The visual difference between the top and bottom AuNPs in the BSE image could be further enhanced with image processing software, such as FIJI/ImageJ. By adjusting the maxima and minima of the black and white threshold, the top nanoparticles changed from spherical to donut shaped, while the bottom nanoparticles remained spherical. This shape difference could be large enough to be used for the automatic registration and differentiation of the markers, which would speed up the process of marker location.

## Discussion

The major issue with the two accelerating voltages, as proposed in publication by Nebesářová et. al.^10^ is that it is necessary to constantly switch them. When the accelerating voltage is switched, it causes the electron beam to defocus and shift to a different position. Returning the beam to the original position and refocusing is time-demanding and increases the radiation damage. We wanted to improve the method in such a way that only one accelerating voltage is used. Since the BSE should have the same energy as the incident beam^11^ and the beam energy decreases by interacting with the ultrathin section, the BSE signal originating from the top NP and bottom NP should be different. This led to the hypothesis that there should be a visual difference between them as well. However, the second thing that needs to be taken into account is the change of the beam diameter caused by scattering. The top NPs are hit by a focused probe, however the bottom ones by inelastically scattered probe with significantly higher broadened diameter–exceeding the diameter of nanoparticle. Together with the different efficiency of the BSE detector for different energy of detected electrons^27^ contribute to the contrast differences in the resulting image.

### The advantage of field emission STEM (FE-STEM)

Is the possibility of working with a low accelerating voltage (up to 30 kV) and obtaining images with more contrast compared to 100 kV TEM at roughly the same resolution^12^. Based on the MC simulation, the electron beam accelerated by 7 kV can still penetrate the 100 nm ultrathin section and provide all the necessary information about the ultrastructure. Moreover, the use of such a low accelerating voltage leads to a significant increase in the image contrast and a decrease in the resolving power^13^. The STEM mode in HRSEM can be used to visualize the cellular structure, comparable with TEM images even without additional postcontrast^14^. This is especially beneficial when visualizing immunolabelled sections, where postfixation by osmium tetroxide is omitted to preserve protein antigenicity and section staining because the additional contrast is undesired as well, as it may “mask” the nanoparticles^15^. In addition, the simultaneous utilization of the BSE mode facilitates the straightforward detection and differentiation of the Au markers. For this reason, finding a suitable accelerating voltage to use both operating modes simultaneously is critical. It has to be high enough to allow the penetration of the BSE signal generated from the Au nanoparticles located at the bottom of the section back to the BSE detector above the sample. On the other hand, it has to be low enough to produce a detectable difference in the appearance of Au NPs enabling their differentiation according to their localization on the section side. The different visibility of the top and bottom NPS is a result of a complicated passage of the beam of primary electrons that undergo inelastic and elastic with the material of the section. These interactions cause beam spreading as well as energy loss. The lower the original accelerating voltage is, the more the beam spreads and the energy of detected electrons is lower.

It is clear that the accelerating voltage and the section thickness represent the most crucial parameters of this method. The section thickness defines what accelerating voltage is needed. The thicker the section is, the higher the accelerating voltage must be to obtain the STEM signal and the BSE signal from the bottom side. We found that for 70 nm ultrathin sections, according to the visual observations, the optimal accelerating voltage to distinguish the top and bottom AuNPs was 4–5 kV, and for 100 nm sections, the optimum was 7–8 kV. The 100 nm sections were used first, to increase the distance and volume of the material between the top and bottom nanoparticles and thus increase our chance to see the visual difference between them. The thicker sections also showed higher stability, as the *C. Velia* cells tend to fall out during or after the sectioning. The thicker section holds them in better. Moreover, increased section thickness improves the image contrast; however, also lowers the resolution^16^ and therefore the procedure was repeated with standard 70 nm sections. If the sections were thinner than 70 nm (e.g., 50 nm) the visualization of the difference between the top and bottom AuNP would require even lower accelerating voltage (< 3 kV). However, the working distance, which would be required for this accelerating voltage is smaller than we can reach with the microscopes (minimal reachable working distance JEOL: 4.8 mm, Apreo + − 7.5 mm).

The STEM detector is always set-up to provide the best possible image contrast and visual difference between the top and bottom markers. This mainly depends on the accelerating voltage, and the section thickness. The tested range was 1–15 kV. At low levels of the accelerating voltage, the intensity of the signal from just BF detector is close to the noise level and the image contrast is minimal. However, when the signal from BF detector is combined with signal from DF or HAADF detectors, we get images with sufficient contrast. The combined image from all three detection fields does not provide additional information or better signal or contrast. The lower accelerating voltages (up to 4 kV with 70 nm sections, up to 6 kV with 100 nm sections) require BF + HAADF detector combination, and the middle values of accelerating voltages (up to 8 and 10 kV) give the best images with a combination of BF and DF detectors. The reason for this is that the electrons accelerated by the low voltage are much more susceptible to scattering, which causes a change in the electron direction; more electrons are detected on the outer rings of the detectors (DF and HAADF). We used the signal from the bright and dark field parts of the STEM detector to achieve the “best” visualization of the whole sample and labelling. Despite we realize that the signals are of different origin, the STEM detector allows to combine the signal from different annuli in the constructive manner. The DF signal provide information about the ultrastructure of uncontrasted specimen, whereas the BF signal gives the information about the AuNPs. The higher accelerating voltages provide sufficient signal and contrast with just BF signal detection, as the scattering is not as large with the higher accelerating voltage. However, these accelerating voltages are already too high to differentiate the AuNPs on the top and bottom (their visual difference is too small or nonexistent).

Low voltage STEM is considered a significant source of radiation damage and causes the contamination of the irradiated area on the ultrathin section surface by the primary electron beam. As explained by Ennos^17^ and Heide^18^, EM contamination is caused by organic molecules that deposit on the specimen and crosslink under electron irradiation. In the modern electron microscopes that are currently used, the specimen chamber vacuum is much cleaner than it was in the past. Therefore, the majority of the organic molecules come from the atmosphere, instead of the vacuum system, as it used to in the past^19^. Strategies for minimizing the contamination in TEM are described in Egerton publication^20^; however, most of these recommendations are also valid for STEM. Generally, plasma cleaner is used very often to suppress contamination^21^. Skoupý et al^22^. studied different parameters, such as section thickness, sample ageing, and dose dependence, on the mass loss of epoxy resin sections. They found that their stability under a 30 kV electron beam was significantly affected by the age of the sections and their thickness. Our results correspond to their findings as the samples that were not plasma cleaned and kept in the vacuum prior to the imaging showed significantly higher build-up of contamination and were more susceptible to the radiation damage.

Thanks to the fact that we can distinguish nanoparticles of the same size/shape/material on the top and bottom sides of the ultrathin section, one should be able to perform, with a carefully chosen sample and antibodies, straightforward multiple labelling. Double labelling would require just one size of marker and therefore could also be quantitatively comparable. It was shown that with increasing NP size, the labelling efficiency decreases (i.e., the 15 nm NPs have much lower efficiency than 5 nm NPs)^2^; therefore, a quantitative comparison of common double labelling using two sizes of NPs is impossible. Another advantage is that both primary antibodies can be from the same species, as cross-reactivity is prevented by the two sides. On the other hand, even though the colocalization of two biomolecules in one organelle is possible, their close interaction cannot be visualized. When two marker sizes are used, quadruple labelling can be achieved. It requires four primary antibodies and two marker sizes (Fig. 3c). This method can be, from our point of view, much easier than attempting to perform multiple labelling in the traditional way. The reason for this is that for traditional multiple labelling, the nanoparticle sizes must be substantially different to avoid ambiguity or be composed of different materials or possess different shapes. This method enables the effective doubling of the number of targets, which can be visualized with the given number of sizes of AuNPs while requiring a single accelerating voltage to record all the images.

## Summary

We showed that the location of immunogold nanoparticles of same size, shape, and material can be clearly distinguished using one single accelerating voltage in the BSE/STEM imaging. This means that with carefully chosen sample one should be able to perform straightforward fast multiple labelling—double labelling requiring just one size of the marker and triple or quadruple two sizes.

## Materials and methods

### Sample preparation

The cells of *Chromera velia* were cultivated as described in Sharaf^23^, harvested by filtering, resuspended in 30 mL of growth medium, and pelleted in Falcon tubes by centrifugation (7910 × g, 5 min, RT). The pellet was then resuspended in 1 ml of growth medium and pelleted in a 1.5 mL microtube by centrifugation (10,000 RPM, 1 min, RT). These pellets were then high-pressure frozen (Leica EM ICE, Leica Microsystems), treated in an automatic freeze substitution unit (Leica EM AFS2, Leica microsystems), embedded in LR-White resin (EMS), and polymerized using UV light. Ultrathin Sects. (70 nm and 100 nm) were cut (Leica EM UMC7, Leica Microsystems) and collected on nickel finder grids (EMS). Both sides of the sections were labelled according to the indirect protocol using rabbit anti-histone H2A primary antibodies (Abcam Cat# ab88770, RRID:AB_10672053) and protein A/secondary antibodies conjugated to either 10 nm and 15 nm AuNPs (CMC PAG10 nm/S, BBI Solutions Cat# EM GAR15/1, RRID:AB_1769134)(Fig. 3a,b). To increase the stability, the grids were coated with a thin layer of carbon (approximately 3 nm) on both sides in a JEOL JEE-4C vacuum evaporator.

### Imaging

Sections were observed using two HRSEMs: 1) Apreo (Thermo Fisher Scientific) using a retractable STEM detector (STEM3 +) and retractable backscatter (CBS) detector with a working distance of approximately 7.5 mm, and JSM-IT800 (JEOL) using a retractable backscatter detector (BED-C) and retractable STEM detector (DEBEN Gen5 ARM2 Annular STEM, DEBEN) with a working distance of 4.8 mm. The tested accelerating voltage ranged from 3 to 15 kV. The samples were plasma cleaned inside the chamber of the SEM prior to imaging to lower the rate of formation of contaminations and decrease the radiation damage.

### Detection of STEM signal

The STEM signal was detected by the detectors operating in annular mode (Fig. 3c). This mode allows a combination of different annuli and the collection of information from a combination of BF, DF, and HAADF. The DF, the combination of BF and DF, and BF and HAADF made it possible to record the images at low accelerating voltages that do not have sufficient signal or have poor image contrast with just BF imaging.

### Monte Carlo simulation

The software used for the MC simulations was a Casino v2.51^24^. Acrylate (C12H22O16) with a density of 0.87832 g/cm^3^ was chosen as a substitute for LR White resin because the exact composition of LR White resin was not available. To simulate our conditions in silico, 3 experiments were designed: a 10 nm gold layer on top of the section and an acrylate layer below it, to simulate gold nanoparticles on the top; a 70 nm acrylate layer on top and a 10 nm gold layer below it, to simulate AuNPs on the bottom of the 70 nm section; and a 100 nm acrylate layer on top and a 10 nm gold layer below it, to simulate AuNPs on the bottom of the 100 nm section (Fig. 3d). For each experiment, there was a simulation composed of fifteen different accelerating voltages (1–15 kV) focused on one spot with the beam radius set to 2 nm. Each simulation was performed with 10 000 000 electrons. All other parameters were unchanged from their default settings. From the resulting distributions of the BSE signal, only 60% of the energies with the highest hit counts were used for the calculations. The broader range was already spread too much to provide relevant data. To compare the energy difference between the top and bottom NPs, we used a median calculated from this 60%.

### Automatic differentiation of top and bottom nanoparticles

The BSE image was uploaded to ImageJ/FIJI software^25^, transformed to 8-bit (if acquired at different bit depths), and filtered twice with a median filter (3 px) to remove the noise. Next, the black and white threshold of the image was adjusted in such a way that the minimum lay on the top tail of the Gaussian distribution, and the exact value corresponds to the Rényi entropy^26^ selection minimal value. The maximum was adjusted manually in such a way that the centres of the top nanoparticles disappeared, and only the ring/donut shape remained.

## Data Availability

The data supporting the findings of this study are available upon reasonable request from the corresponding author F.K.
